# Histologically confirmed case of cerebral vasculitis associated with Crohn’s disease –a case report

**DOI:** 10.1186/s12883-015-0429-7

**Published:** 2015-09-21

**Authors:** Masayuki Gekka, Taku Sugiyama, Masafumi Nomura, Yasutaka Kato, Hiroshi Nishihara, Katsuyuki Asaoka

**Affiliations:** Department of Neurosurgery, Teine Keijinkai Medical Center, 1-40 Maeda 1-12, Teine-Ku, Sapporo, 006-8555 Japan; Center for Gastroenterology, Teine Keijinkai Medical Center, 1-40 Maeda 1-12, Teine-Ku, Sapporo, 006-8555 Japan; Department of Translational Pathology, Hokkaido University Graduate School of Medicine, North 15, West 7, Kita-ku, Sapporo, 060-8638 Japan

**Keywords:** Central nervous system, Crohn’s disease, Inflammatory bowel disease, Neurological involvement, Vasculitis

## Abstract

**Background:**

Extraintestinal manifestations in Crohn’s disease (CD) are frequent and well recognized. However, neurological involvement secondary to CD is rare, and there have been few histologically confirmed cases of cerebral vasculitis secondary to CD.

**Case presentation:**

A 58-year-old left-handed man with a history of refractory CD who had fever of over 38 °C, progression of CD symptoms, and Gerstmann’s syndrome consulted our hospital. Laboratory data showed elevation of C-reactive protein (CRP) and hypoproteinemia. T2-weighted magnetic resonance imaging (MRI) revealed a right parietal high-intensity lesion. Catheter angiography showed segmental multiple narrowing and occlusion in the distal part of the middle cerebral artery and anterior cerebral artery. Angiography also revealed multiple venous occlusions in the affected parietal area. To confirm the diagnosis, the patient underwent open biopsy, and histological examination revealed cerebral vasculitis. The patient was then started on high-dose prednisolone (60 mg/day) in addition to his previous therapy, which included mesalazine, adalimumab, and azathioprine. CRP elevation, hypoproteinemia, and gastrointestinal symptoms immediately improved after starting this treatment. Neurological status improved simultaneously with CD symptom improvement, and follow-up brain MRI revealed a reduction in the size of the right parietal lobe lesion. He returned to normal status and was discharged from our hospital 5 weeks after admission.

**Conclusion:**

This is an important case of histologically confirmed cerebral vasculitis associated with CD. The clinical course of our case clearly illustrates the relevance of the occurrence of cerebral vasculitis and the exacerbation of CD.

## Background

Crohn’s disease (CD), an autoimmune disorder, is an increasingly prevalent chronic inflammatory bowel disease (IBD) that may affect any part of the gastrointestinal tract from mouth to anus. Extraintestinal manifestations occur frequently in the joints, skin, eyes, and coagulation system in IBD patients [1, 2].

Recent studies indicate that neurological manifestations in IBD appear to be more common than previously estimated [3–5], but this is not yet widely recognized. Nevertheless, early recognition and treatment are crucial in preventing major morbidity in patients with neurological involvement. In contrast, although cerebral vasculitis associated with ulcerative colitis has been relatively well described in the literature [6–10], cerebral vasculitis secondary to CD has been reported in only a few anecdotal case reports [11–16], and there have been few histologically confirmed cases.

Here we describe a histologically confirmed case of cerebral vasculitis associated with CD. The occurrence of cerebral vasculitis was closely correlated with exacerbation of CD, and early treatment led to simultaneous improvement in both neurological and CD symptoms.

## Case presentation

**Fig. 1:**
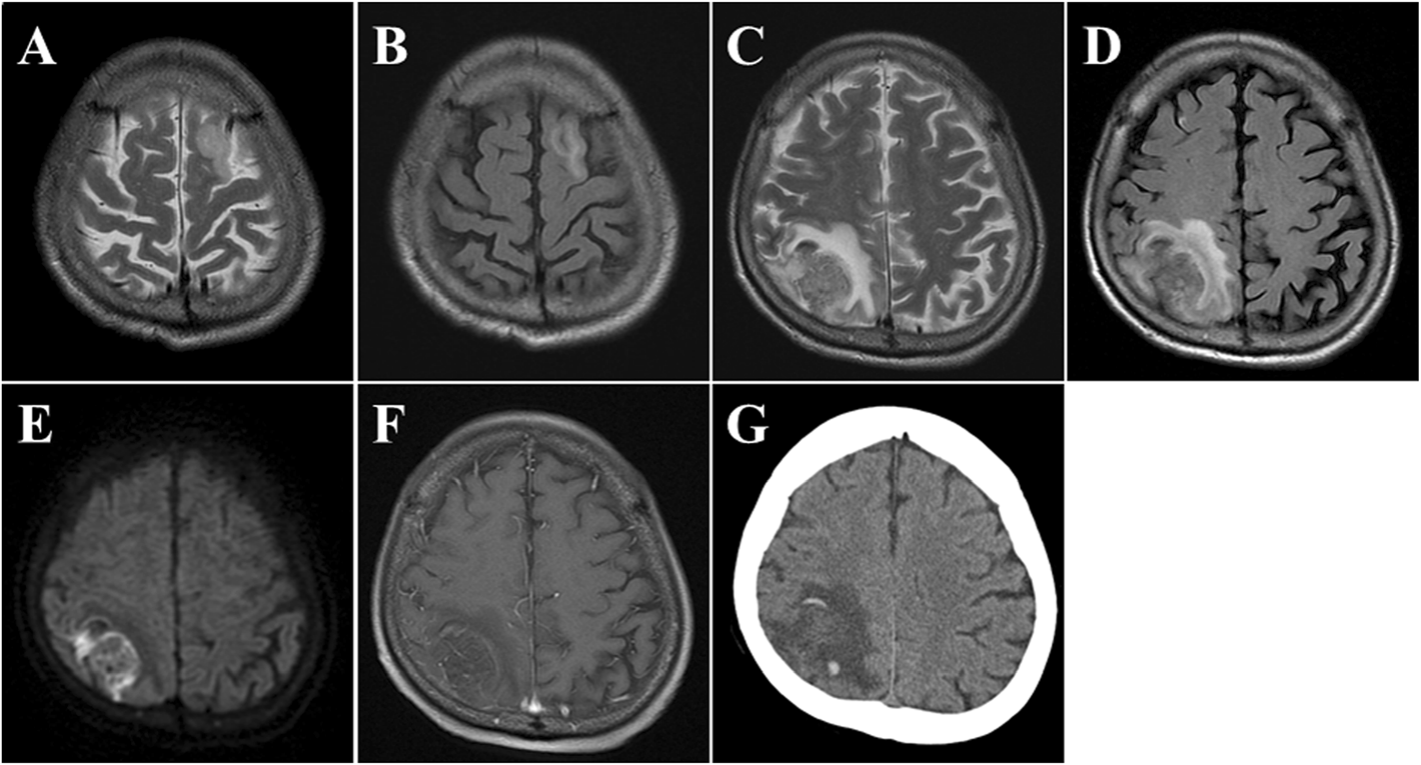
T2 weighted magnetic resonance imaging (MRI) (T2WI) and fluid-attenuated inversion recovery (FLAIR) 3 months before admission revealed a white matter lesion in the left superior frontal gyrus (**a**, **b**). T2WI and FLAIR on admission revealed a right parietal and frontal white matter high intensity lesion (**c**, **d**). Diffusion MRI showed a high-intensity lesion in the right parietal gray matter (**e**), and T1-weighted MRI after gadolinium administration revealed no enhancing structures (**f**). CT revealed high density area in the right parietal and frontal lesion (**g**)

Here we present the case of a 58-year-old left-handed man who was admitted to our hospital. He had chronic diarrhea, hematochezia, and hypoproteinemia, and was diagnosed as having CD on the basis of the endoscopic and histological findings at 51 years of age . He had been treated with mesalazine (5-ASA), adalimumab (ADA), azathioprine (AZA), and supplemental corticosteroids at times of active disease, but he had experienced refractory CD symptoms since the onset of the disease. Three months before his consultation, he had an episode of transient loss of consciousness. T2-weighted magnetic resonance imaging (MRI) (T2WI) and fluid-attenuated inversion recovery (FLAIR) revealed a white matter lesion in the left superior frontal gyrus (Fig. 1a, b). He underwent conservative medical treatment, because he had no general symptoms and no neurological findings to date. This lesion disappeared on follow-up MRI 1 week later.

He developed difficulty writing words 3 days before admission, and had fever of over 38 °C with frequent diarrhea and hematochezia, which suggested exacerbation of CD symptoms. On admission to our hospital, the patient complained of acalculia, agraphia, apraxia, finger agnosia, left–right disorientation, and his neurological status rapidly deteriorated with left-side hemiparesis within the following 48 h. Laboratory data showed elevated C-reactive protein (CRP, 3.73 mg/dL) and hypoproteinemia, in the form of decreased total protein (3.5 g/dL) and albumin (1.4 g/dL). Complete blood count, blood glucose, renal function tests, and serum electrolytes were normal. Serological tests including rheumatoid factor, antinuclear antibodies, anti-double-stranded DNA, antineutrophil cytoplasmic antibodies, rapid plasma regain/Venereal Disease Research Laboratory slide test, antiphospholipid antibodies, and tumor markers were negative. Infectious workups including Hepatitis B and C virus, Human Immunodeficiency virus, Influenza A and B virus, Toxoplasmosis, Tuberculosis, and *Treponema pallidum* were also negative. Lumbar puncture revealed that cerebrospinal fluid (CSF) was clear and colourless, and opening pressure was 130 mm H2O. His CSF was normal (cell count, <1/μL; glucose, 72 mg/dL; protein, 16 mg/dL; Cl, 121 mEq/L; myelin basic protein, 61.8 pg/mL; IgG, 2.8 mg/dL; IgA, 0.4 mg/dL; IgM, 0.5 mg/dL; IgG index, 0.3; oligoclonal band negative), and negative for CSF culture. Polymerase chain reaction test for Herpes simplex viruses 1 and 2, Varicella-zoster virus, and Cytomegalo virus were also negative. Total body computed tomography (CT) excluded malignancy but showed edema of the terminal ileum, suggesting active CD. Chest X-ray and CT showed bilateral pleural effusion, suggesting an association with hypoproteinemia.

**Fig. 2:**
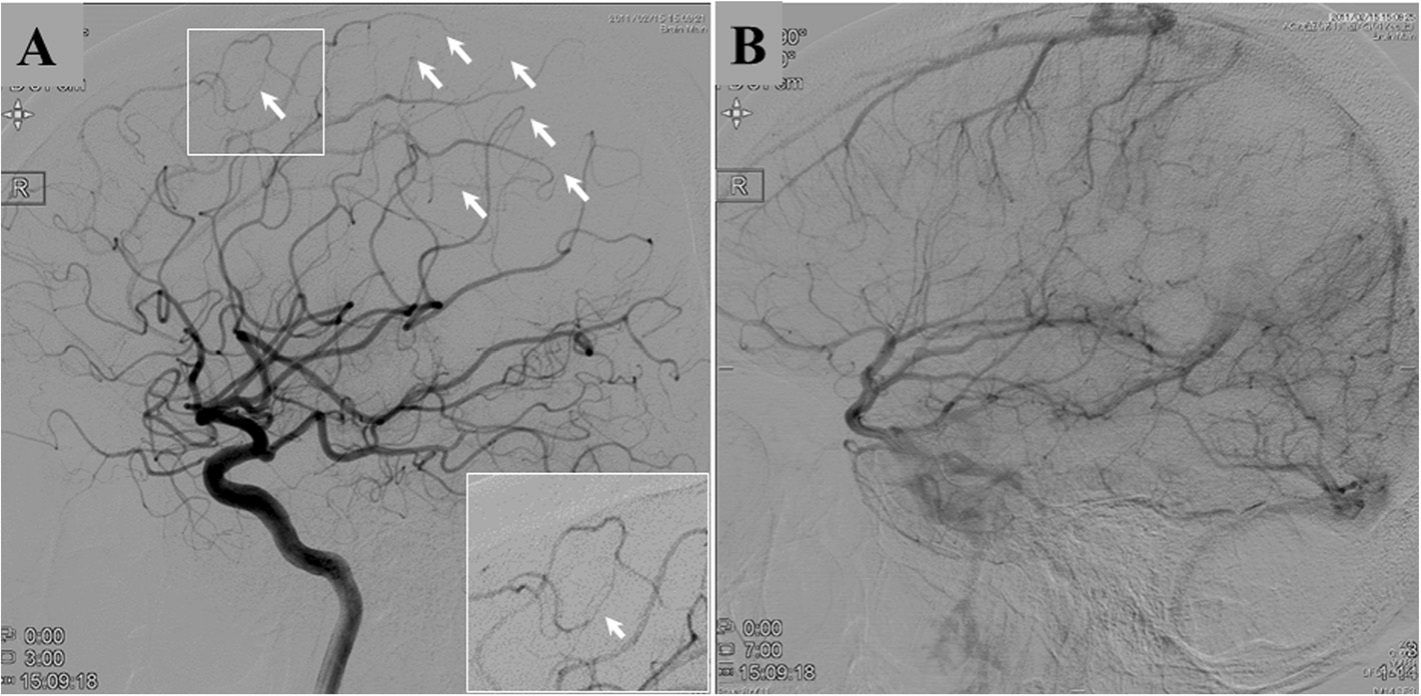
Lateral view of right internal carotid angiography of arterial phase (**a**) revealed segmental multiple narrowing (*arrows*) of the distal part of the anterior cerebral artery and middle cerebral artery. The venous phase of angiography (**b**) revealed multiple venous occlusions in the right parietal area

Head CT revealed a low-density lesion in the right parietal lobe and a hemorrhagic high-density area inside the lesion. T2WI, FLAIR, and diffusion-weighted MRI revealed a right parietal abnormal high-intensity lesions that did not contain a gadolinium-enhanced area on T1-weighted MRI (Fig. 1c-g). Magnetic resonance angiography revealed no pathological findings, but catheter angiography of the right carotid artery showed multifocal narrowing and occlusion of the distal portion of the right anterior cerebral artery and middle cerebral artery (Fig. 2a). The venous phase of angiography also revealed multiple cortical venous occlusions in the affected right parietal area (Fig. 2b).

**Fig. 3:**
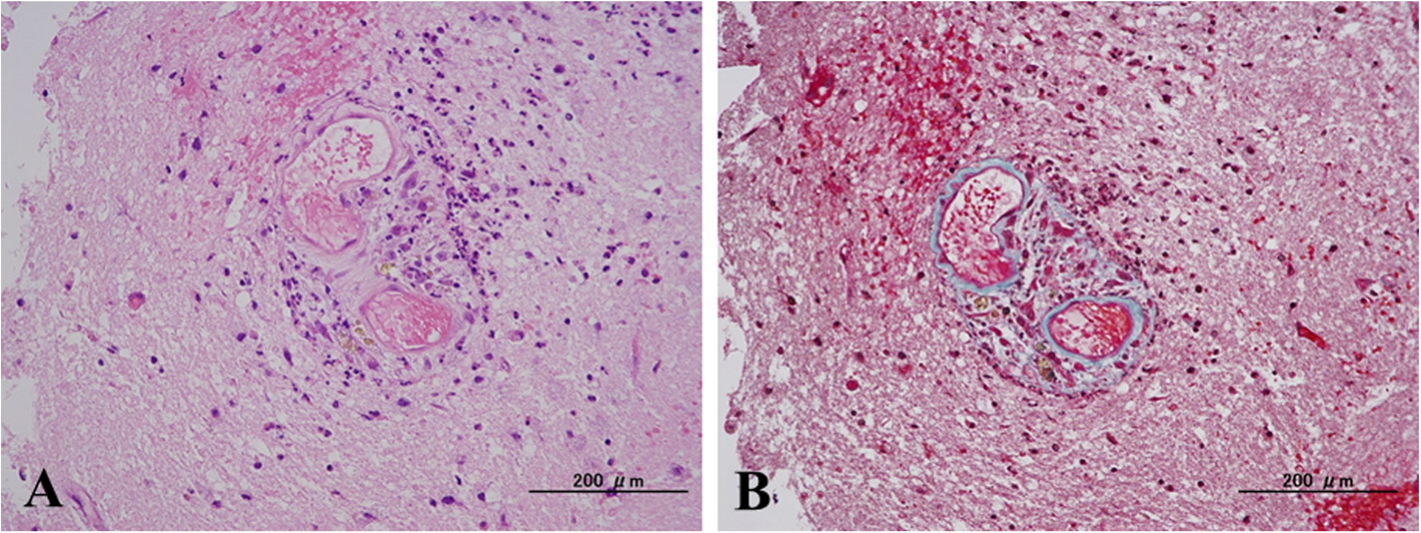
Hematoxylin-Eosin (**a**) and Elastica-Masson staining (**b**) of the specimen. Neutrophils, eosinophils, and histocyte infiltrate within the thickend vessel wall of the cortical artery. Hemorrhage around the perivascular area can be seen

To confirm the diagnosis and exclude other inflammatory diseases, open biopsy was then performed. A small craniotomy directly over the clinically affected parietal area revealed localized subarachnoid hemorrhaging and venous thrombosis. Approximately 1 cm^3^ edematous brain tissue including the cortex with a longitudinally oriented surface vessel, white matter, and overlying leptomeninges was obtained as a specimen. Microscopic examination (hematoxylin–eosin staining) showed inflammatory infiltrates in the vessel wall with extravasation of red blood cells. Inflammatory infiltrates included neutrophils, eosinophils and histiocytes. These findings were compatible with vasculitis, although fibrinoid necrosis was not demonstrated (Fig. 3).

**Fig. 4:**
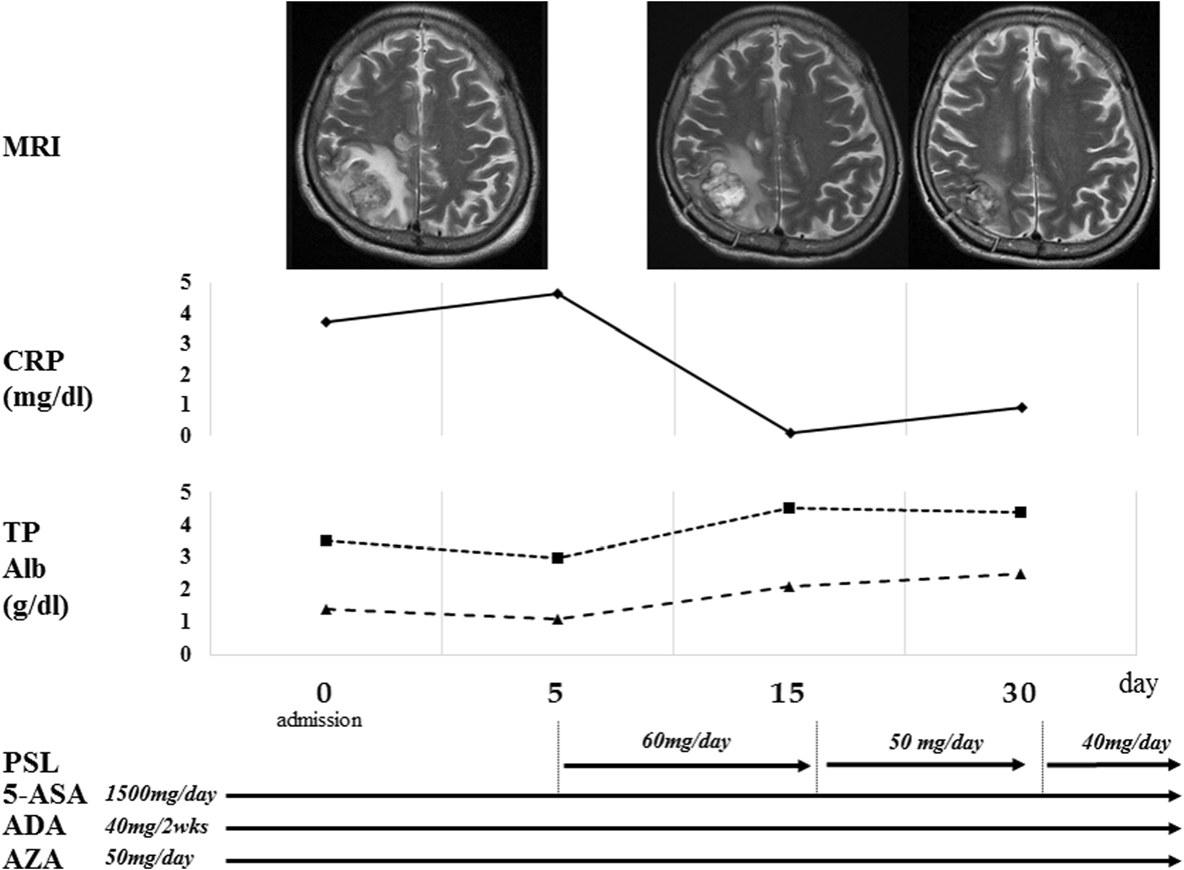
Course of medication, laboratory data, and follow up T2WI. 5-ASA, mesalazine; ADA, adalimumab; AZA, azathioprine; and PSL, prednisolone

**Fig. 5:**
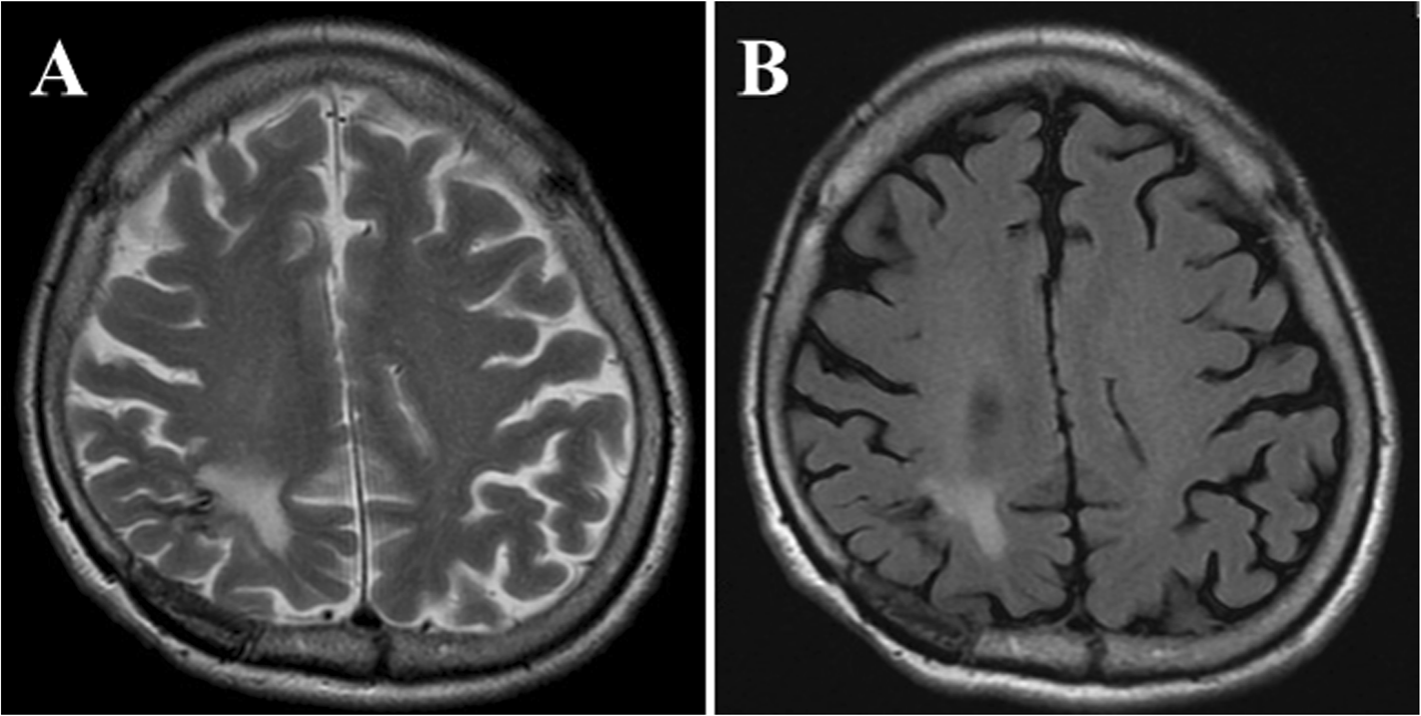
T2WI (**a**) and FLAIR (**b**) 4 years after admission revealed no new lesion

The patient was then started on high-dose prednisolone (60 mg/day) in addition to the previous treatment, which included 5-ASA, ADA, and AZA. His CRP elevation, hypoproteinemia, bilateral pleural effusion, fever, and gastrointestinal symptoms immediately improved. Moreover, simultaneously with the improvement of his CD symptoms, his neurological status improved, with a reduction of the right parietal lobe lesion on follow-up MRI (Fig. 4). He gradually regained normal functional status and was discharged from our hospital 5 weeks after admission without neurological symptoms. No new neurological symptoms and MRI abnormalities appeared in the subsequent 4-year period (Fig. 5).

## Conclusions

In this case report we describe a histologically confirmed case of cerebral vasculitis associated with CD. Interestingly, the occurrence of cerebral vasculitis showed a good correlation with exacerbation of the patient’s CD, and additional corticosteroid therapy improved both neurological and CD symptoms simultaneously.

To date, several types of neurological disorders including cerebral infarction [3, 17], cerebral venous thrombosis [18], demyelinating central nervous system (CNS) disease [19, 20], and peripheral neuropathy [21], have been reported. Importantly, cerebral infarction was the most commonly reported neurological complication of IBD. These thrombotic complications are thought to be attributed to hypercoagulability and are two to four times more likely to occur in patients with IBD than in healthy individuals [1]. IBD was associated with an increase in the risk if ischemic or hemorrhagic stroke or transient ischemic attack (OR = 1.28, 95 % CI: 1.17-1.41) [22]. It is estimated that 1.3 % to 6.4 % of adults with IBD and 3.3 % of children with IBD develop cerebrovascular complications sometime in the course of their disease [3, 23]. In MRI studies, asymptomatic focal white matter hyperintensity lesions were frequently observed in IBD patients (42 % of patients with CD and 46 % of patients with UC) [24, 25]. A recent study revealed that, compared with healthy control subjects, in patients with IBD there was a larger number of white matter hyperintensities associated with gray matter volume loss in multiple regions in the temporal, frontal, and parietal cortex and decreased axial diffusivity values in major white matter tracts [4]. Several possible pathogenetic mechanisms, including cerebral infarction, cerebral vasculitis, and direct neurotoxic effects of inflammatory cytokines have been considered, but the actual mechanism remains uncertain. Accumulation of more cases is essential to clarify the mechanism. The diagnosis of secondary cerebral vasculitis is sometimes difficult because of the heterogeneous clinical presentations and lack of a specific diagnostic laboratory test and/or imaging test [26]. MRI typically reveals multifocal and bilateral gray and white matter lesions in 75 % of these patients, but this is not a pathognomonic finding in cerebral vasculitis. Catheter angiography typically reveals alternating stenosis and ectasia with or without aneurysms of multiple vessels in multiple vascular beds, but these angiographic findings can be observed in several other inflammatory and non-inflammatory conditions, which may have different treatments [27]. Brain biopsy is still the gold standard for diagnosis of cerebral vasculitis, although it is an invasive procedure [28]. Consensus statements on therapeutic strategies for cerebral vasculitis secondary to CD do not exist because of its rarity. In the present case, the patient had already been treated with 5-ASA, ADA, and AZA, and we therefore started corticosteroid therapy in addition to the immunosuppressive treatment. Fortunately, we obtained a good result in this case. Appropriate diagnosis and early treatment seem to be important in patients with cerebral vasculitis secondary to CD. However, accumulation of more cases is essential before an appropriate diagnostic and therapeutic strategy can be established.

### Consent

Written informed consent was obtained from the patient for publication of this Case report and any accompanying images. A copy of the written consent is available for review by the Editor of this journal.

## Abbreviations

5-ASA: Mesalazine
ADA: Adalimumab
AZA: Azathioprine
CD: Crohn’s disease
CNS: Central nervous system
CRP: C-reactive protein
CT: Computed tomography
IBD: Inflammatory bowel disease
MRI: Magnetic resonance imaging
T2WI: T2-weighted imaging
